# Serosurvey of peste des petits ruminants virus in small ruminants from different agro-ecological zones of Nigeria

**DOI:** 10.4102/ojvr.v83i1.1035

**Published:** 2016-03-11

**Authors:** Timothy Y. Woma, Pius S. Ekong, Dauda G. Bwala, John O. Ibu, Louisa Ta’ama, Dyek Y. Dyek, Ladi Saleh, David Shamaki, Demo J.U. Kalla, Dalan Bailey, Haruna M. Kazeem, Melvyn Quan

**Affiliations:** 1 Department of Veterinary Tropical Diseases, University of Pretoria, South Africa; 2 Morbilliviruses Research Laboratory, National Veterinary Research Institute, Nigeria; 3 Animal Production Programme, Abubakar Tafawa Balewa University, Nigeria; 4 School of Immunity and Infection, University of Birmingham, United Kingdom; 5 Department of Veterinary Microbiology, Ahmadu Bello University Zaria, Nigeria

## Abstract

Peste des petits ruminants, caused by the peste des petits ruminants virus (PPRV), is a highly contagious and economically important transboundary viral disease of domestic and wild small ruminants and a major hindrance to small-ruminant production in Nigeria. The seroprevalence and distribution of PPRV antibodies in small ruminants in rural households, farms, live animal markets and slaughter slabs across the six different agro-ecological zones of Nigeria were determined. A total of 4548 serum samples from 3489 goats and 1059 sheep were collected in 12 states. A PPRV competitive enzyme-linked immunosorbent assay was used to test the samples and the data analysed with R statistical software version 3.0.1. The study animals included all ages and both sexes. The overall prevalence estimate of sera positive for PPRV antibodies was 23.16% (*n* = 1018 positive samples per 4548 total samples, 95% confidence interval: 21.79% – 24.57%). There were significant differences in the seroprevalence between the states (*p* = 0.001). Taraba State had the highest seroprevalence of 29.51%, whilst the lowest seroprevalence of 14.52% was observed in Cross River State. There were no significant differences in the PPRV seroprevalence between male and female animals (*p* = 0.571), age (*p* = 0.323) and between species (*p* = 0.639). These data indicate the current seroprevalence to PPRV in the small-ruminant population in Nigeria.

## Introduction

Peste des petits ruminants (PPR) is a highly contagious and economically important transboundary viral disease of domestic and wild small ruminants (Balamurugan *et al.* 2010). Outbreaks of PPR occur regularly in small ruminants throughout Nigeria and are characterised by pyrexia, depression, anorexia, diarrhoea, respiratory distress, mucopurulent oculo-nasal discharge with matting of the eyelids, necrotic oral lesions that produce a foetid smell and sometimes abortion in pregnant animals. The disease is caused by the peste des petits ruminants virus (PPRV), which is classified in the genus *Morbillivirus* within the family *Paramyxoviridae* (King *et al.* 2012).

There are many documented reports of the endemic nature of PPRV in Nigeria (El-Yuguda *et al.* 2010; Emikpe & Akpavie 2010; Ezeibe *et al.* 2008; Ibu *et al.* 2008; Isoun & Mann 1972; Obi *et al.* 1983; Obidike *et al.* 2006; Odo 2003; Ogunsanmi *et al.* 2003; Okoli 2003; Shamaki 2002; Taylor & Abegunde 1979; Ularamu *et al.* 2012). Outbreaks have continued to occur in the country during the last 40 years, despite the introduction of the tissue culture rinderpest vaccine against PPRV in the 1980s and the use of the homologous PPRV vaccine (Diallo 2003; Diallo *et al.* 1989) since 1998. The National Veterinary Research Institute Vom, Nigeria, produces a 50-dose PPRV vaccine vial using the Nigeria 75/1 strain, with the recommendations that small ruminants must be vaccinated from 3 months of age and thereafter every 12 months.

Small ruminants play an important role in agricultural food production and in sustainable employment in Nigeria and the control and eradication of PPR is therefore a priority, in order to ease poverty and improve the health and husbandry of animals kept by resource-poor people in this developing country. This study was designed to determine the seroprevalence and distribution of PPRV antibodies in small ruminants in rural households, farms, live animal markets and slaughter slabs across different states in all the agro-ecological zones of Nigeria. This information will be helpful to develop a progressive control programme with the aim to eradicate PPR from Nigeria.

## Materials and methods

### Study area

Each agro-ecological zone consists of administrative structures called States, subdivided into local governments. Two states were selected at random from each agro-ecological zone: the north-eastern agro-ecological zone is montane savannah and samples were collected from Adamawa and Taraba States; the south-eastern agro-ecological zone is tropical rain forest and samples were collected from Anambra and Imo States; the south-southern agro-ecological zone is mangrove or swamp and samples were collected from Akwa Ibom and Cross River States; the north-central agro-ecological zone is Guinea or derived savannah and samples were collected from Kwara and Plateau States; the north-western agro-ecological zone is Sudan and Sahel savannah and samples were collected from Kano and Sokoto States; the south-western agro-ecological zone is tropical rain forest and samples were collected from Ogun and Ondo States (Iloeje 2001).

### Sample size and sample collection

The OpenEpi v2 open-source calculator, SSPropor (Kevin, Andrew & Minn 2009) was used to calculate the total number of samples to be collected per state using the equation:

Sample size (*n*) = DEFF × *Np*(1 − *p*)/[*d*^2^/*Z*^2^_1 − α/2_ × (*N* − 1) + *p*(1 − *p*)] [Eqn 1]

where DEFF = design effect for cluster surveys, *N* = population size, *p* = hypothesised % frequency of outcome factor in a population and *d* = confidence limits as ± %. The calculated sample size per state was 379 (Table 1).

**TABLE 1:** Apparent and true prevalence of peste des petits ruminants virus antibodies in small ruminants in Nigeria, 2010–2013.

Category	Region	Number of samples analysed	Number positive	Apparent prevalence (%)	TP (%)	*X*^2^	*p*
State	Adamawa	379	76	20.05	20.52	30.9	0.001
	Taraba	379	106	27.97	29.51	-	-
	Anambra	379	74	19.53	19.92	-	-
	Imo	379	102	26.91	28.31	-	-
	AkwaIbom	379	94	24.80	25.91	-	-
	Cross River	379	56	14.76	14.52	-	-
	Plateau	379	79	20.84	21.41	-	-
	Kwara	379	101	26.65	28.01	-	-
	Kano	379	108	28.49	30.11	-	-
	Sokoto	379	84	22.16	22.91	-	-
	Ogun	379	67	17.68	17.82	-	-
	Ondo	379	71	18.73	19.02	-	-
Sex	Male animals	1925	421	21.87	22.58	0.321	0.571
	Female animals	2623	597	22.76	23.59	-	-
Species	Sheep	1059	244	23.04	23.91	0.220	0.639
	Goat	3489	774	22.18	22.93	-	-
Age	Adult	3040	664	21.38	22.55	0.977	0.323
	Young	1508	354	23.48	24.41	-	-
**Total**		**4548**	**1018**	**22.38**	**23.16**	**-**	**-**

The combined population of domestic small ruminants in Nigeria in 2007 was estimated at 90 million (FAOSTAT 2007). Nigeria has a 36-state structure, and a figure of 2 000 000 small ruminants per state was estimated for sample size calculations (in effect an infinite population). A percentage frequency of outcome factor in the population was hypothesised to be 44% ± 5% based on a study by Shamaki (2002). A confidence limit of 5% and design effect of one was used.

Multistage sampling was performed with four hierarchical stages. The first level of selection was the state. Two states were selected randomly from each agro-ecological zone of the country. Within each state, local governments, districts and villages were selected randomly. Within each selected village, animals were sampled from different homes, from the live animal market and from the slaughter slabs. These were selected purposely because of logistical constraints.

About 5 mL of blood was collected by venipuncture. Sera were separated within 24 h after centrifugation at a relative centrifugal force of 1000 for 10 min. All samples were then stored at -20 °C until used.

### Competitive enzyme-linked immunosorbent assay

The commercially available competitive enzyme-linked immunosorbent assay (c-ELISA) kit (IDVET, France) for PPRV was used for this study. This diagnostic kit detects antibodies directed against the nucleoprotein of PPRV and was developed by a FAO reference laboratory (CIRAD-EMVT, Montpellier, France). The microplates were supplied in strips, already precoated with PPRV recombinant nucleoprotein. The kit contained anti-Nucleoprotein Horseradish peroxidase (NP-HRP) concentrated conjugate (10×), positive and negative controls, dilution buffers, wash concentrate (20×), substrate solution and stop solution (0.5 M H_2_SO_4_). The test was performed according to the manufacturer’s instructions (Libeau *et al.* 1995). The ELISA microplates were read with an immunoscan reader (Flow Laboratories, UK) with a filter of 450 nm. The optical density (OD) was recorded and the test was validated when the mean value of the negative control OD (ODNC) was greater than 0.7 and the mean value of the positive control OD was less than 0.3 of the ODNC.

The OD values were converted to competition percentage (CP) using the following formula: CP = OD sample/ODNC × 100. The samples with CP 35% (cut-off) were considered positive for PPRV infection.

### Statistical analysis

The overall and group apparent prevalence (total positive/total sample analysed) and the exact binomial 95% confidence intervals (CI) were computed. Pearson’s chi-squared test was used to determine if there were significant differences in the number of positive and negative test results within each variable. A *p* < 0.05 was considered significant for all tests. The true prevalence (TP) together with 95% CI for all categories was calculated using the Rogan–Gladen adjusted estimator of ‘true’ prevalence (Rogan & Gladen 1978). TP was calculated using the following equation:

TP = (AP + Sp − 1) / (Se + Sp − 1) [Eqn 2]

where AP = apparent prevalence, Sp = specificity and Se = sensitivity. The sensitivity of the c-ELISA test has been reported to be 90% and specificity 98% (Saliki *et al.* 1993). All analyses were performed in R statistical software version 3.0.1 (R 2015).

## Results

**FIGURE 1:**
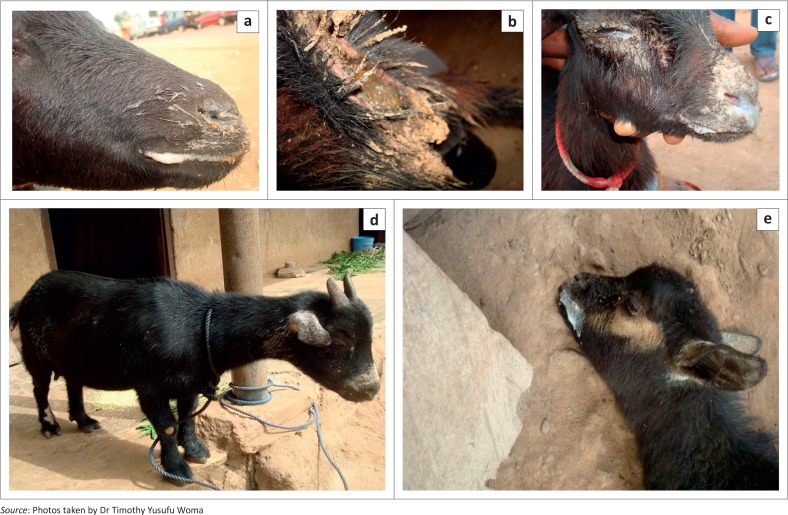
Clinical signs (compatible with Peste des petits ruminants) observed during sampling (2010–2013) in various locations in Nigeria: (a) frothy salivation and muco-purulent nasal discharge; (b) soiled anal region because of profuse diarrhoea; (c) matted eyelids, salivation and nasal discharges; (d) raised hair coat, depression and increased rectal temperature and (e) frothy salivation and death.

**FIGURE 2:**
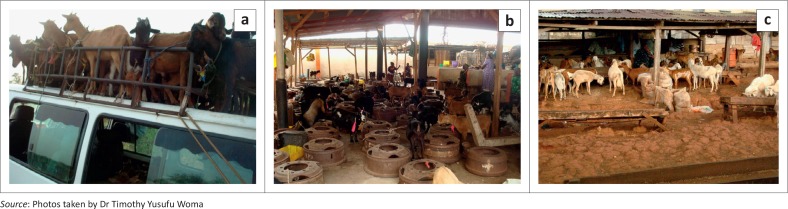
(a) Small ruminants were transported in an overcrowded vehicle from the north-eastern part of the country to the south. Note the exposure to weather elements (sun, rain) in addition to the stress of transportation over long distances without feed or water. (b and c) Live animal market in a south-western Nigerian city.

At a 95% confidence level, a sample size of 379 animals per state was calculated. A total of 4548 field serum samples were collected from 12 states across the agro-ecological zones of the country. No animal was known to have been vaccinated against PPRV before or at the time of sampling. Some animals appeared to have clinical signs compatible with PPR during sampling (Figure 1a–e). Small ruminants were transported in vehicles that were often overcrowded, over long distances from the north of the country to the live animal markets in the south (Figure 2a). On arrival at the markets, the animals were tied together as they awaited buyers (Figure 2b).

The overall apparent prevalence estimate of sera positive for PPRV antibodies was 22.38% (*n* = 1018/4548, 95% CI: 21.79–24.57), with a range of 14.5% – 30.1%. The overall TP estimate was 23.16% (Table 1).

**FIGURE 3:**
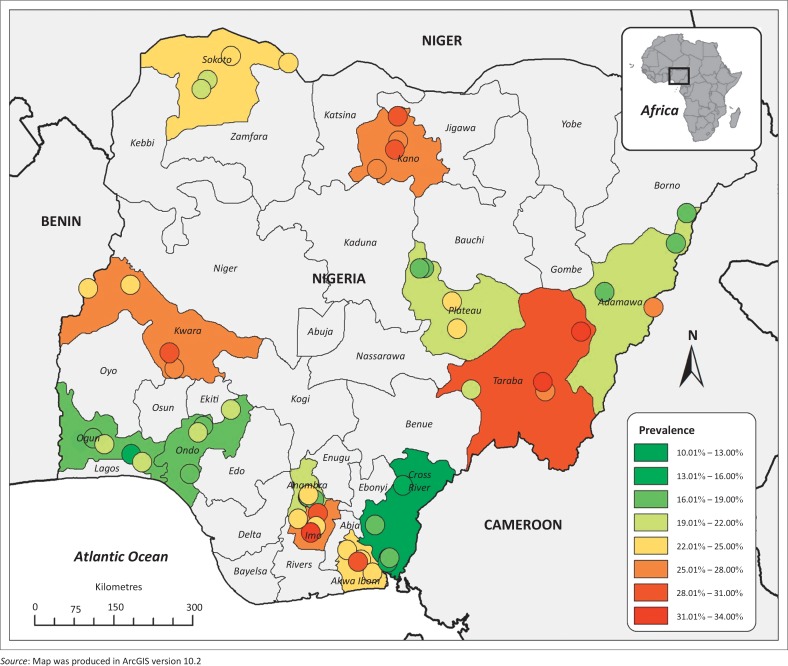
Prevalence of peste des petits ruminants virus antibodies in each locality indicated by coloured circles. The colour of each state indicates the overall prevalence in that state.

**FIGURE 4:**
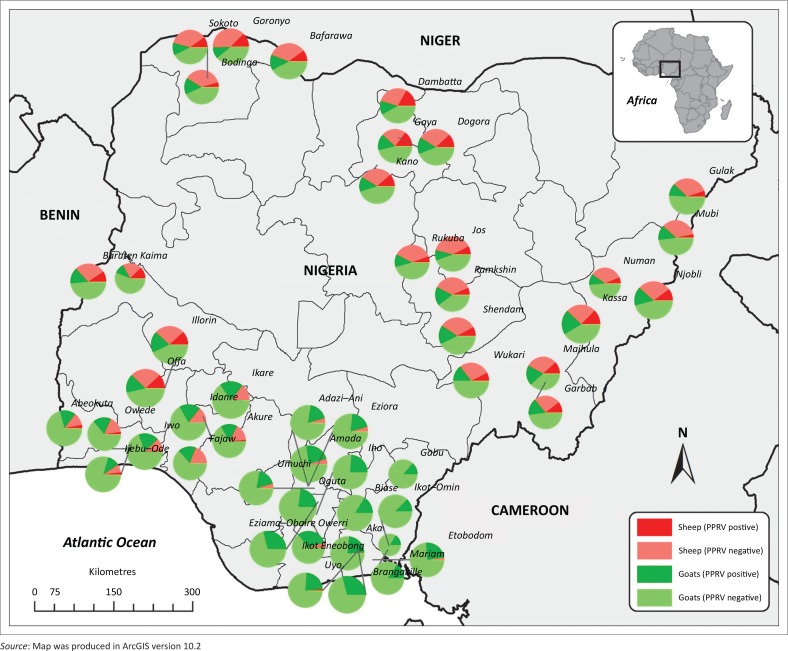
The proportion of sheep and goats seropositive to peste des petits ruminants virus in Nigeria. The size of the pie symbol is relative to the number of samples collected.

There were significant differences in the distribution of PPRV antibodies in small ruminants between states (*p* = 0.001) (Table 1, Figure 3). The highest seroprevalence was in Taraba State (29.51%), whilst the lowest seroprevalence was observed in Cross River State (14.52%). The highest seroprevalence of PPRV in sheep where *n* > 6 was in Kano State (33.12%, *n* = 157) and in goats it was in Taraba State (27.97%, *n* = 236) (Figure 4). The seroprevalence varied between localities, with a range of 12.64% (Ikot-Omin, Cross River State) to 33.33% (Kassa, Taraba State) (Figure 4). No spatial pattern was evident in the distribution of PPRV antibodies in small ruminants – the seroprevalence did not appear to be higher in states neighbouring other countries, nor higher in one region of the country.

From the 4548 small ruminants that were sampled, 42.3% were male animals and 57.7% were female animals (Table 1). True prevalence was 22.58% for male animals and 23.59% for female animals. There was no significant difference in the PPRV seroprevalence between male and female small ruminants (*p* = 0.571).

The animals that were sampled ranged in age from 3 months to 3 years. The TP of PPRV in animals above 1 year of age was 22.55% and 24.40% in animals between 3 and 12 months of age. There was no significant difference between the seroprevalence of PPRV in small ruminants over 1 year of age and those between 3 and 12 months of age (*p* = 0.323) (Table 1).

A total of 3489 serum samples were collected form goats and 1059 serum samples from sheep. The TP in goats was 22.93% and it was 23.91% in sheep. There was no significant difference between the seroprevalence of PPRV in sheep and goats (*p* = 0.639) (Table 1).

The sample size in each locality varied from 43 (Ikot Eneobong, Cross River State) to 111 (Kassa, Taraba State) with a median of 95 (Figure 4). In most localities in the south of Nigeria, sheep were seronegative for PPRV. In this region, the sample sizes in sheep were either small (Akwa Ibom, Anambra and Imo States) or no sheep were available for sampling (Cross River State), as most farmers breed goats and sheep are scarce. However, in Idanre and Fajaw, both in Ondo State, all 14 and 17 sheep, respectively, were seronegative for PPRV. The highest seroprevalence of PPRV in sheep in a locality where the sample size was greater than two was in Gaya, Kano State (38.24%, *n* = 34). The largest number of sheep sampled in a locality (*n* = 44) was in Bafarawa (Sokoto State), where the seroprevalence of antibodies to PPRV in sheep was 22.73%.

Goats were present in all localities sampled, with a range of 43 (Ikot Eneobong, Cross River State) to 104 (Brangaville, Akwa Ibom State). The prevalence in goats ranged from 12.36% (Ijebu-Ode, Ogun State) to 34.00% (Maihula, Taraba State) (Figure 4).

The seroprevalence of PPRV antibodies in each animal category was very similar (20.44% in male adult sheep, 22.29% in female adult sheep, 21.64% in young male sheep, 23.78% in young female sheep, 23.89% in adult male goats, 22.09% in adult female goats, 26.21% in young male goats and 24.02% in young female goats). The highest seroprevalence was 66.67% in young female sheep from Kwara State (*n* = 18).

## Discussion

Analysis of 4548 samples from sheep and goats confirmed the endemic nature and wide distribution of PPRV throughout Nigeria. This study found an overall current seroprevalence of PPRV antibodies in small ruminants in Nigeria to be 23.16%. Taylor and Abegunde (1979) in the 1970s reported a seroprevalence of 57% and 44% in sheep and goats respectively. Obi *et al.* (1983) reported 52.2% and 53.7% in sheep and goats in the 1980s, whilst Shamaki (2002) reported a seroprevalence of 44% from samples analysed between 1995 and 1999. These differences may be because of the sampling locations, sample frames, seasons of the year, different sampling techniques and laboratory tests used. It is also possible that the higher seroprevalence obtained previously was because of sampling during outbreaks. The worldwide eradication of rinderpest virus, which cross-reacts with PPRV in serological tests, may also have been responsible for the lower seroprevalence we found in this study.

These results are similar to a study in Ethiopia, which reported a seroprevalence of 33% in sheep and 67% in goats in the 1990s, but another study a decade later found the seroprevalence to be 9% in goats and 13% in sheep (Abraham *et al.* 2005; Roeder *et al.* 1994). The difference was less in Sudan (51.9% compared to 67.2%) in studies conducted 10 years apart (Haroun *et al.* 2002; Saeed *et al.* 2010).

Taraba State in the north-east agro-ecological zone had the highest seroprevalence rate of 29.51%, whilst Cross River State in the south-southern agro-ecological zone had the lowest seroprevalence rate of 14.52%. This observation may be because of the prevailing management practices in the various states and rainfall, which affects the growth of grasses. Most of the small ruminants in Taraba State are on a free range (agro-pastoralism) system because the farmlands for crops are at a distance from most villages and the zone is a savannah with a longer dry season period. Most animals in Cross River State are tied at home and grass is provided because crops are planted in the backyard. Cross River State is a rain forest zone with abundant rain that enables grass to grow almost all the year round. The sheep breeds observed in Taraba State were of the Uda, Balami and Yankassa breeds, whilst those seen in Cross River State were predominantly of the West African dwarf (WAD) stock. The WAD breeds of goats were also seen in Cross River State, while those in TarabaState were a mix of both the WAD and the Sokoto Red. It is not clear whether these breed differences in small ruminants had any effect on the difference in seroprevalence rates observed in the two states.

Transportation, management practices and marketing play major roles in the epidemiology of PPRV. We observed that animals from different households and localities are usually transported together in close proximity (Figure 2a) to the market. At the market, the animals are tied together (Figure 2b) in stalls as they await buyers. Any animal not sold is returned to the flock and new animals bought also end up in new flocks. These practices are conducive to outbreaks of diseases. New infections leading to outbreaks normally occur when animals are transported together or when they are tied together (transportation stress, overcrowding and malnutrition) in the live animal markets or kept for safety reasons with a relative from another locality.

In this study, there was an over-representation of female animals (2623 female animals and 1925 male animals). This may be because of the practice of sacrificing male animals when the need to sell or slaughter animals arises.

The c-ELISA is an ideal test to carry out a serological survey of PPRV antibodies in small ruminants. The c-ELISA was used in this study because of the merits it has over other serological techniques, which are more laborious and time consuming.

The results of this study may have been biased by the sampling technique employed. Because of the logistical constraints and the complexity of the pastoral husbandry practices, it was not possible to systematically select the different villages, farms, households and the animals to include in this study. Live sheep and goat markets and slaughter slabs were also sampled randomly in each locality visited. A more accurate seroprevalence may emerge if randomised sampling is carried out where the actual small-ruminant population in each state (data such as these are currently not available in Nigeria) is taken into account.

An important observation during the course of sampling is the apparent lack of awareness amongst the small-ruminant owners of the vaccination status of their flocks. The control and eradication of PPR is a priority in order to ease poverty and improve the health and husbandry of animals kept by people in developing countries. It is encouraging to note that between 18 and 23 November 2013, heads of veterinary laboratories and directors of veterinary services from the ECOWAS subregion gathered in Lagos to develop a framework for the progressive control of PPR in the region. This type of subregional approach also took place in southern Africa and a PanAfrican strategy for the progressive control of PPRV has also been developed (Elsawalhy *et al.* 2010).

This study provides an overview of the current antibody seroprevalence to PPRV in the small-ruminant population in Nigeria and also confirms a low level of awareness about vaccination amongst small-holder farmers in rural settings.

## Appendix 1

**TABLE 1-A1:** Small-ruminant peste des petits ruminants virus seroprevalence survey from different localities in Nigeria, 2010–2013.

Province	Town	Latitude	Longitude	Sheep positive	Sheep negative	Sheep collected	Goat positive	Goat negative	Goat collected	Total positive	Total Collected	Prev_sheep	Prev_goats	Prev_Total
Adamawa State	Gulak	10.806	13.456	5	32	37	11	50	61	16	98	13.51	18.03	16.33
Adamawa State	Mubi	10.300	13.267	3	33	36	13	45	58	16	94	8.33	22.41	17.02
Adamawa State	Njobli	9.227	12.883	11	31	42	17	50	67	28	109	26.19	25.37	25.69
Adamawa State	Numan	9.483	12.050	5	26	31	9	38	47	14	78	16.13	19.15	17.95
Akwa Ibom State	Aka	5.183	7.650	0	1	1	20	68	88	20	89	0.00	22.73	22.47
Akwa Ibom State	Brangaville	4.983	7.833	0	1	1	30	74	104	30	105	0.00	28.85	28.57
Akwa Ibom State	Etobodom	4.800	8.083	0	2	2	22	68	90	22	92	0.00	24.44	23.91
Akwa Ibom State	Uyo	5.017	7.883	1	0	1	21	71	92	22	93	100.00	22.83	23.66
Anambra State	Adazi-Ani	6.064	6.973	0	4	4	17	72	89	17	93	0.00	19.10	18.28
Anambra State	Amada	6.102	6.987	0	5	5	23	76	99	23	104	0.00	23.23	22.12
Anambra State	Eziora	6.070	6.986	0	4	4	18	73	91	18	95	0.00	19.78	18.95
Anambra State	Umuchi	6.025	7.084	0	4	4	16	71	87	16	91	0.00	18.39	17.58
Cross Rivers State	Biase	5.602	8.121	0	0	0	16	82	98	16	98	-	16.33	16.33
Cross Rivers State	Gabu	6.267	8.600	0	0	0	9	58	67	9	67	-	13.43	13.43
Cross Rivers State	Ikot Eneobong	5.056	8.372	0	0	0	7	36	43	7	43	-	16.28	16.28
Cross Rivers State	Ikot-Omin	5.050	8.357	0	0	0	11	76	87	11	87	-	12.64	12.64
Cross Rivers State	Mariam	4.975	8.337	0	0	0	13	71	84	13	84	-	15.48	15.48
Imo State	Eziama-Obaire	5.797	7.144	0	0	0	29	71	100	29	100	-	29.00	29.00
Imo State	Iho	5.577	7.115	0	0	0	22	70	92	22	92	-	23.91	23.91
Imo State	Oguta	5.705	6.806	1	1	2	23	78	101	24	103	50.00	22.77	23.30
Imo State	Owerri	5.477	7.025	1	3	4	26	54	80	27	84	25.00	32.50	32.14
Kano State	Dambatta	12.426	8.509	16	27	43	12	40	52	28	95	37.21	23.08	29.47
Kano State	Dogora	12.013	8.521	12	29	41	15	44	59	27	100	29.27	25.42	27.00
Kano State	Gaya	11.867	8.467	13	21	34	14	41	55	27	89	38.24	25.45	30.34
Kano State	Kano	11.539	8.158	11	28	39	13	43	56	24	95	28.21	23.21	25.26
Kwara State	Baruten	9.550	3.231	9	27	36	14	47	61	23	97	25.00	22.95	23.71
Kwara State	Illorin	8.474	4.619	12	27	39	19	44	63	31	102	30.77	30.16	30.39
Kwara State	Kaima	9.604	3.947	9	15	24	8	41	49	17	73	37.50	16.33	23.29
Kwara State	Offa	8.217	4.700	13	29	42	17	51	68	30	110	30.95	25.00	27.27
Ogun State	Abeokuta	7.050	3.317	3	11	14	14	68	82	17	96	21.43	17.07	17.71
Ogun State	Ijebu-Ode	6.783	3.967	2	7	9	11	78	89	13	98	22.22	12.36	13.27
Ogun State	Iwo	6.650	4.150	2	10	12	18	69	87	20	99	16.67	20.69	20.20
Ogun State	Owode	6.948	3.505	2	14	16	15	55	70	17	86	12.50	21.43	19.77
Ondo State	Akure	7.253	5.192	1	14	15	14	58	72	15	87	6.67	19.44	17.24
Ondo State	Fajaw	6.450	4.967	0	17	17	15	56	71	15	88	0.00	21.13	17.05
Ondo State	Idanre	7.151	5.109	0	14	14	19	66	85	19	99	0.00	22.35	19.19
Ondo State	Ikare	7.533	5.667	1	15	16	21	68	89	22	105	6.25	23.60	20.95
Plateau State	Jos	9.882	8.969	7	37	44	9	42	51	16	95	15.91	17.65	16.84
Plateau State	Pankshin	9.327	9.443	6	33	39	16	36	52	22	91	15.38	30.77	24.18
Plateau State	Rukuba	9.882	8.885	5	34	39	11	42	53	16	92	12.82	20.75	17.39
Plateau State	Shendam	8.877	9.527	8	33	41	17	43	60	25	101	19.51	28.33	24.75
Sokoto State	Bafarawa	13.317	6.650	10	34	44	14	42	56	24	100	22.73	25.00	24.00
Sokoto State	Bodinga	12.875	5.167	4	33	37	14	39	53	18	90	10.81	26.42	20.00
Sokoto State	Goronyo	13.433	5.667	12	37	49	10	38	48	22	97	24.49	20.83	22.68
Sokoto State	Sokoto	13.033	5.267	9	33	42	11	39	50	20	92	21.43	22.00	21.74
Taraba State	Garbabi	7.838	11.035	9	22	31	14	41	55	23	86	29.03	25.45	26.74
Taraba State	Kassa	8.822	11.643	14	28	42	23	46	69	37	111	33.33	33.33	33.33
Taraba State	Maihula	7.989	10.995	10	26	36	17	33	50	27	86	27.78	34.00	31.40
Taraba State	Wukari	7.866	9.765	7	27	34	14	48	62	21	96	20.59	22.58	21.88
**Total**	**-**	**-**	**-**	**101**	**377**	**478**	**170**	**489**	**659**	**271**	**93.5**	**21.15**	**25.59**	**23.74**

**TABLE 2-A1:** Nigerian individual state peste des petits ruminants virus seroprevalence data based on species of small ruminants, 2010–2013.

Province	SheepPos	SheepNeg	SheepTotal	Prev_sheep	GoatsPos	GoatsNeg	GoatTotal	Prev_goats	TotalPos	TotalNeg	Total	Prevalence
Anambra	0	13	13	0.00	74	292	366	20.22	74	305	379	19.53
Akwa Ibom	1	7	8	12.50	94	277	371	25.34	95	284	379	24.80
Adamawa	24	122	146	16.44	52	181	233	22.32	76	303	379	20.05
Imo	2	4	6	33.33	100	273	373	26.81	102	277	379	26.91
Taraba	40	103	143	27.97	66	170	236	27.97	106	273	379	28.50
Plateau	26	137	163	15.95	53	163	216	24.54	79	300	379	20.84
Kano	52	105	157	33.12	54	168	222	24.32	106	273	379	27.97
Ogun	9	42	51	17.65	58	270	328	17.68	67	312	379	17.68
Kwara	43	95	138	31.16	58	183	241	24.07	101	278	379	26.65
Ondo	2	60	62	3.23	69	248	317	21.77	71	308	379	18.73
Cross River	0	0	0	-	56	323	379	14.78	56	323	379	14.78
Sokoto	35	137	172	20.35	49	158	207	23.67	84	295	379	22.16

**TABLE 3-A1:** Peste des petits ruminants virus seroprevalence data based on category of small ruminants in Nigeria, 2010–2013.

Province	SMA pos	SMY pos	SFA pos	SFY pos	GMA pos	GMY pos	GFA pos	GFY pos	SMA neg	SMY neg	SFA neg	SFY neg	GMA neg	GMY neg	GFA neg	GFY neg	Prev_SMA	Prev_GMA	Prev_SMY	Prev_GMY	Prev_SFA	Prev_GFA	Prev_SFY	Prev_GFY
Anambra	0	0	0	0	17	12	24	21	8	0	5	0	69	66	85	73	0.00	19.77	-	15.38	0.00	22.02	-	22.34
Akwa Ibom	1	0	0	0	11	23	36	24	3	0	3	1	31	61	120	65	25.00	26.19	-	27.38	0.00	23.08	0.00	26.97
Adamawa	8	3	12	1	15	5	18	14	48	11	52	11	54	22	71	34	14.29	21.74	21.43	18.52	18.75	20.22	8.33	29.17
Imo	1	0	1	0	29	18	32	21	1	0	3	0	73	56	77	67	50.00	28.43	-	24.32	25.00	29.36	-	23.86
Taraba	13	6	16	5	21	7	26	14	36	11	40	16	50	17	72	29	26.53	29.58	35.29	29.17	28.57	26.53	23.81	32.56
Plateau	8	2	12	4	16	7	21	9	45	12	57	23	48	17	68	30	15.09	25.00	14.29	29.17	17.39	23.60	14.81	23.08
Kano	14	8	21	9	14	9	19	12	32	15	40	18	38	27	70	33	30.43	26.92	34.78	25.00	34.43	21.35	33.33	26.67
Ogun	5	0	3	1	11	8	27	12	14	2	21	5	50	25	69	26	26.32	18.03	0.00	24.24	12.50	28.13	16.67	31.58
Kwara	11	4	16	12	21	9	23	5	32	12	45	6	68	25	68	22	25.58	23.60	25.00	26.47	26.23	25.27	66.67	18.52
Ondo	1	0	1	0	13	8	32	16	17	11	20	12	71	49	81	47	5.56	15.48	0.00	14.04	4.76	28.32	0.00	25.40
Cross River	0	0	0	0	11	18	23	4	0	0	0	0	76	73	120	54	-	12.64	-	19.78	-	16.08	-	6.90
Sokoto	12	4	13	6	14	5	22	8	52	12	58	15	45	17	70	26	18.75	23.73	25.00	22.73	18.31	23.91	28.57	23.53
**Total**	**74**	**27**	**95**	**38**	**193**	**129**	**303**	**160**	**288**	**86**	**344**	**107**	**673**	**455**	**971**	**506**	**20.44**	**23.89**	**21.64**	**26.21**	**22.29**	**22.09**	**23.78**	**24.02**
